# Prognostic significance of chronic kidney disease and impaired renal function in Japanese patients with COVID-19

**DOI:** 10.1186/s12879-024-09414-w

**Published:** 2024-05-25

**Authors:** Hiromu Tanaka, Shotaro Chubachi, Takanori Asakura, Ho Namkoong, Shuhei Azekawa, Shiro Otake, Kensuke Nakagawara, Takahiro Fukushima, Ho Lee, Mayuko Watase, Kaori Sakurai, Tatsuya Kusumoto, Katsunori Masaki, Hirofumi Kamata, Makoto Ishii, Naoki Hasegawa, Yukinori Okada, Ryuji Koike, Yuko Kitagawa, Akinori Kimura, Seiya Imoto, Satoru Miyano, Seishi Ogawa, Takanori Kanai, Koichi Fukunaga

**Affiliations:** 1 Division of Pulmonary Medicine, Department of Medicine, Keio University School of Medicine, 35 Shinanomachi, Shinjuku, Tokyo, 160-8582 Japan; 2 Department of Clinical Medicine (Laboratory of Bioregulatory Medicine), Kitasato University School of Pharmacy, Tokyo, Japan; 3 Department of Respiratory Medicine, Kitasato University, Kitasato Institute Hospital, Tokyo, Japan; 4 Department of Infectious Diseases, Keio University School of Medicine, Tokyo, Japan; 5 Department of Respiratory Medicine, Nagoya University Graduate School of Medicine, Nagoya, Japan; 6 Department of Statistical Genetics, Osaka University Graduate School of Medicine, Suita, Japan; 7 Department of Genome Informatics, Graduate School of Medicine, the University of Tokyo, Tokyo, Japan; 8 Laboratory for Systems Genetics, RIKEN Center for Integrative Medical Sciences, Kanagawa, Japan; 9 Health Science Research and Development Center (HeRD), Tokyo Medical and Dental University, Tokyo, Japan; 10 Department of Surgery, Keio University School of Medicine, Tokyo, Japan; 11 Institute of Research, Tokyo Medical and Dental University, Tokyo, Japan; 12 Division of Health Medical Intelligence, Human Genome Center, the Institute of Medical Science, the University of Tokyo, Tokyo, Japan; 13 M&D Data Science Center, Tokyo Medical and Dental University, Tokyo, Japan; 14 Department of Pathology and Tumor Biology, Kyoto University, Kyoto, Japan; 15 Division of Gastroenterology and Hepatology, Department of Internal Medicine, Keio University School of Medicine, Tokyo, Japan

**Keywords:** Renal insufficiency, COVID-19, Japanese population, Estimated glomerular filtration rate

## Abstract

**Background:**

Renal impairment is a predictor of coronavirus disease (COVID-19) severity. No studies have compared COVID-19 outcomes in patients with chronic kidney disease (CKD) and patients with impaired renal function without a prior diagnosis of CKD. This study aimed to identify the impact of pre-existing impaired renal function without CKD on COVID-19 outcomes.

**Methods:**

This retrospective study included 3,637 patients with COVID-19 classified into three groups by CKD history and estimated glomerular filtration rate (eGFR) on referral: Group 1 (*n* = 2,460), normal renal function without a CKD history; Group 2 (*n* = 905), impaired renal function without a CKD history; and Group 3 (*n* = 272), history of CKD. We compared the clinical characteristics of these groups and assessed the effect of CKD and impaired renal function on critical outcomes (requirement for respiratory support with high-flow oxygen devices, invasive mechanical ventilation, or extracorporeal membrane oxygen, and death during hospitalization) using multivariable logistic regression.

**Results:**

The prevalence of comorbidities (hypertension, diabetes, and cardiovascular disease) and incidence of inflammatory responses (white blood counts, and C-reactive protein, procalcitonin, and D-dimer levels) and complications (bacterial infection and heart failure) were higher in Groups 2 and 3 than that in Group 1. The incidence of critical outcomes was 10.8%, 17.7%, and 26.8% in Groups 1, 2, and 3, respectively. The mortality rate and the rate of requiring IMV support was lowest in Group 1 and highest in Group 3. Compared with Group 1, the risk of critical outcomes was higher in Group 2 (adjusted odds ratio [aOR]: 1.32, 95% confidence interval [CI]: 1.03–1.70, *P* = 0.030) and Group 3 (aOR: 1.94, 95% CI: 1.36–2.78, *P* < 0.001). Additionally, the eGFR was significantly associated with critical outcomes in Groups 2 (odds ratio [OR]: 2.89, 95% CI: 1.64–4.98, *P* < 0.001) and 3 (OR: 1.87, 95% CI: 1.08–3.23, *P* = 0.025) only.

**Conclusions:**

Clinicians should consider pre-existing CKD and impaired renal function at the time of COVID-19 diagnosis for the management of COVID-19.

**Supplementary Information:**

The online version contains supplementary material available at 10.1186/s12879-024-09414-w.

## Background

The coronavirus disease (COVID-19) pandemic has caused several epidemic waves and resulted in a shortage of hospital beds and strain on the medical care system in Japan, especially in metropolitan areas. Thus, effective strategies are urgently needed for predicting and preventing severe COVID-19.

Various patient characteristics and laboratory findings are associated with COVID-19 severity [1–5]. Renal impairment, including chronic kidney disease (CKD) and acute kidney injury (AKI), is a known predictor of COVID-19 severity [1, 6]. A systematic review found that COVID-19 mortality is higher among patients with CKD, and that mortality was proportional to CKD severity [7]. In Japan, the COVID-19 mortality rate is higher among patients undergoing dialysis than that in the general population [8]. CKD is a risk factor for AKI in patients with COVID-19, and COVID-19 mortality is higher in patients with AKI [9]. A small study in Japan found that renal function and urinary findings during hospitalization were associated with COVID-19 severity [10]. However, a reliable clinical diagnosis of CKD is often difficult, requiring laboratory data prior to COVID-19. Therefore, although many studies have defined CKD based on the renal function at the time of COVID-19 diagnosis, to our knowledge, none have assessed the impact of pre-existing CKD, or impaired renal function without pre-existing CKD, on the clinical outcomes in patients with COVID-19.

This study assessed the impact of a history of CKD or poor renal function on referral on the critical outcomes of Japanese patients with COVID-19.

## Methods

### Study design and setting

This retrospective cohort study used data collected by the Japan COVID-19 Task Force [11], a nationwide multicenter consortium [12]. All patients provided written or oral informed consent, and the study was approved by the ethics committee of the Keio University School of Medicine (20,200,061) and related research institutions. A flow chart of the study design is shown in Additional file Fig. S1. From February 2020 to August 2022, 4,171 patients with COVID-19 confirmed using SARS-CoV-2 polymerase chain reaction or antigen tests were recruited, of whom 534 were excluded due to the following exclusion criteria: non-Japanese (*n* = 110); incomplete renal function data (*n* = 68); oxygen requirement at referral (*n* = 13); or no data for evaluating history of CKD (*n* = 135). Additionally, we excluded 208 patients who required high-flow oxygen devices, invasive mechanical ventilation (IMV), or extracorporeal membrane oxygenation support at referral, leaving 3,637 patients in the analysis.

### Data collection and definitions

The data were collected on age, sex, body mass index (BMI), smoking history, medical history (including hypertension, diabetes mellitus, cardiovascular disease, and CKD), symptoms and signs, laboratory data, radiographic imaging findings, oxygen support at referral, complications after referral noted by the physician in charge (including bacterial infection, heart failure, thromboembolism, and AKI), and clinical outcome. The physician in charge interviewed patients about their medical history, including CKD history, to identify patients with a history of CKD. The symptoms and signs included those reported at referral and those observed during hospitalization. Laboratory data, including serum creatinine concentrations, were collected within 48 h of referral. Rapid spread of shadow on chest radiography was defined as enlargement to more than 50% of the entire lung field within 48 h of referral. The kidney function at baseline was determined based on the estimated glomerular filtration rate (eGFR), calculated using age, sex, and serum creatinine concentration collected within 48 h of referral, as previously defined for the Japanese population [13]. An AKI complication was defined as a worsening of renal function and other complications, based on the judgement of the physician in charge. The critical outcome was defined as requiring respiratory support with high-flow oxygen devices, IMV, or extracorporeal membrane oxygenation, or death during hospitalization, as previously described [3, 5].

We classified the patients into three groups based on their history of CKD and eGFR on referral as follows: Group 1 (*n* = 2,460), normal renal function (eGFR ≥ 60 mL/min/1.73 m^2^) without CKD; Group 2 (*n* = 905), impaired renal function (eGFR < 60 mL/min/1.73 m^2^) without CKD; Group 3 (*n* = 272), CKD history. Laboratory data prior to referral were not available and the history of CKD relied on the patient report.

### Statistical analysis

Continuous variables were compared using independent sample t-tests, and categorical variables were compared using chi-square tests. A multivariable logistic regression adjusted for age </≥ 65 years; sex; BMI </≥ 25 kg/m^2^; smoking history; and medical history of hypertension, diabetes mellitus, or cardiovascular disease was used to evaluate the relationship between each group and a critical outcome [2, 3, 14, 15]. Two-tailed *P-*values < 0.05 were considered statistically significant. For comparisons among Groups 1, 2, and 3, we used a Bonferroni-adjusted significance level of *P* < 0.0167 (0.05/3). All statistical analyses were conducted using JMP 16 (SAS Institute Japan Ltd., Tokyo, Japan). Visualization was performed using GraphPad Prism version 8.0 (GraphPad Software, San Diego, CA, USA) and the R Bioconductor package *ggalluvial*.

## Results

### Clinical characteristics

Additional file Fig. S2 shows the distribution of eGFR for the 3,637 patients. The median (interquartile range) for each group was as follows: Group 1, 78.2 (69.4–90.7) mL/min/1.73 m^2^; Group 2, 51.1 (43.2–55.9) mL/min/1.73 m^2^; Group 3, 29.4 (8.3–43.5) mL/min/1.73 m^2^. Based on the previously defined grades for CKD [16], Group 3 included 137 patients (50.4%) with CKD grades 4–5 (eGFR < 30 mL/min/1.73 m^2^), 31 (11.4%) patients undergoing dialysis, and five (1.8%) kidney transplant recipients. Table 1 shows the clinical characteristics of each group. Patients in Groups 2 and 3 were older and had a higher prevalence of comorbidities, including hypertension, diabetes, and cardiovascular disease, than those in Group (1) Patients in Group 3 were older, more likely to be male, and had a higher prevalence of comorbidities than those in Group (2) There were no significant differences in systemic symptoms, such as fever and fatigue, among the three groups. However, the incidence of upper respiratory tract symptoms, such as dysgeusia and dysosmia, was lower, and the incidence of lower respiratory tract symptoms, such as cough and shortness of breath, was higher in Groups 2 and 3 than in Group 1.

**Table 1:** Comparison of clinical characteristics among the three patient groups according to renal function status

Parameters	Normal ranges	All (*n* = 3,637) All patients	Group 1 (*n* = 2,460) History of CKD (−) Impaired renal function (−)	Group 2 (*n* = 905) History of CKD (−) Impaired renal function (+)	Group 3 (*n* = 272) History of CKD (+)	*P* value
Age [years]		58 (45–72)	52 (40–66)	69 (57–78)^***^	71 (59–79)^***^	< 0.001
Sex, male		2415 (66.4)	1604 (65.2)	606 (67.0)	205 (75.4)^***,##^	0.003
BMI [kg/m^2^]		23.9 (21.4–27.0)	23.8 (21.3–27.0)	24.0 (21.8–26.8)	24.2 (21.5–-27.3)	0.998
Smoking history						0.027
Never		1784 (52.6)	1243 (54.1)	420 (49.8)	121 (47.8)	
Previously or currently		1609 (47.4)	1053 (45.9)	424 (50.2)	132 (52.2)	
Medical history						
Hypertension		1267 (35.1)	586 (24.0)	485 (53.8)^***^	196 (72.9)^***,###^	< 0.001
Diabetes mellitus		746 (20.7)	391 (16.0)	224 (25.1)^***^	131 (48.5)^***,###^	< 0.001
Cardiovascular disease		413 (11.4)	158 (6.5)	149 (16.6)^***^	106 (39.0)^***,###^	< 0.001
Malignancy		300 (8.3)	176 (7.2)	88 (9.8)^*^	36 (13.5)^***^	< 0.001
Autoimmune disease		189 (5.2)	118 (4.8)	42 (4.7)	29 (10.8)^***,###^	< 0.001
COPD		172 (4.8)	93 (3.8)	55 (6.2)^**^	24 (9.0)^***^	< 0.001
Asthma		253 (7.1)	184 (7.6)	56 (6.3)	13 (4.9)	0.137
Hyperuricemia		359 (10.0)	138 (5.7)	141 (15.8)^***^	80 (29.6)^***,###^	< 0.001
Chronic liver disease		166 (4.6)	105 (4.3)	38 (4.2)	23 (8.5)^**,##^	0.006
Dialysis		31 (0.9)	0 (0.0)	0 (0.0)	31 (11.4)^***,###^	< 0.001
Kidney transplant		5 (0.1)	0 (0.0)	0 (0.0)	5 (1.8)^***,###^	< 0.001
Immunosuppressive therapy^a)^		177 (4.9)	105 (4.3)	37 (4.1)	35 (13.0)^***,###^	< 0.001
Symptoms						
Fever (≥ 37.5℃)		2932 (81.3)	1983 (81.3)	741 (82.6)	208 (77.0)	0.120
Cough		2296 (63.9)	1592 (65.4)	553 (62.1)	151 (56.3)^**^	0.006
Sputum		1110 (30.6)	743 (30.6)	292 (32.9)	75 (28.2)	0.279
Sore throat		967 (27.1)	690 (28.6)	213 (23.9)^**^	64 (24.1)	0.013
Rhinorrhea		517 (14.5)	373 (15.5)	110 (12.4)	34 (12.8)	0.063
Dysgeusia		628 (17.6)	485 (20.0)	125 (14.1)^***^	18 (6.7)^***,##^	< 0.001
Dysosmia		527 (14.7)	426 (17.6)	85 (9.6)^***^	16 (6.0)^***^	< 0.001
Shortness of breath		1329 (37.4)	866 (36.0)	365 (41.2)^**^	98 (37.3)	0.023
Fatigue		1966 (54.9)	1300 (53.6)	515 (57.8)	151 (56.6)	0.086
Laboratory data						
WBC count [/µL]	3300–8600	5100 (4000–6700)	4980 (3900–6500)	5470 (4100–7000)^***^	5440 (4230–7280)^***^	< 0.001
Neutrophil [/µL]	1800–7500	3530 (2560–5050)	3380 (2460–4860)	3890 (2760–5390)^***^	3940 (2940–5610)^***^	< 0.001
Lymphocyte [/µL]	1000–4800	960 (680–1320)	1000 (710–1370)	900 (660–1250)^***^	800 (590–1180)^***,#^	< 0.001
Albumin [g/dL]	4.1–5.1	3.8 (3.3–4.2)	3.9 (3.4–4.2)	3.6 (3.3–4.0)^***^	3.4 (2.9–3.8)^***,###^	< 0.001
LDH [IU/L]	124–222	248 (194–346)	236 (186–336)	274 (216–382)^***^	258 (204–329)	< 0.001
UA [mg/dL]	3.7–7.0	4.7 (3.7–5.9)	4.4 (3.5–5.5)	5.4 (4.3–6.5)^***^	6.1 (4.9–7.3)^***,##^	< 0.001
HbA1c [%]	4.9–5.9	6.0 (5.6–6.5)	5.9 (5.6–6.4)	6.1 (5.8–6.8)^***^	6.1 (5.7–7.1)^***^	< 0.001
Ferritin [ng/mL]	42–326	376 (174–732)	366 (163–722)	433 (223–821)^***^	315 (125–597)^#^	< 0.001
KL-6 [U/mL]	< 500	241 (180–350)	226 (172–328)	281 (198–408)^***^	286 (211–427)^***^	< 0.001
PCT [ng/mL]	< 0.04	0.07 (0.04–0.13)	0.05 (0.03–0.09)	0.10 (0.06–0.19)^***^	0.25 (0.08–0.65)^***^	< 0.001
CRP [mg/dL]	< 0.14	3.17 (0.79–7.72)	2.53 (0.56–6.84)	4.91 (1.89–9.45)^***^	3.95 (1.10–9.35)^***^	< 0.001
D-dimer [µg/mL]	< 0.90	0.91 (0.51–1.40)	0.80 (0.50–1.30)	1.00 (0.60–1.70)^**^	1.30 (0.80–2.38)^***^	< 0.001
eGFR [ml/min/1.73 m^2^]		69.9 (55.3–84.0)	78.2 (69.4–90.7)	51.1 (43.2–55.9)^***^	29.4 (8.3–43.5)^***,###^	< 0.001
Chest X-ray						
GGO [bilateral/unilateral]		1875 (53.6)/402 (11.5)	1190 (50.7)/285 (12.1)	539 (60.7)/89 (10.0)^***^	146 (55.5)/28 (10.7)	< 0.001
Consolidation [bilateral/unilateral]		757 (21.8)/334 (9.6)	485 (20.9)/206 (8.9)	209 (23.8)/99 (11.3)^*^	63 (24.0)/29 (11.0)	0.031
Rapid spread of the shadow		337 (10.4)	199 (9.1)	103 (12.6)^**^	35 (14.1)^*^	0.002
Chest CT						
GGO [bilateral/unilateral]		2326 (70.1)/320 (9.6)	1500 (67.7)/221 (10.0)	648 (76.4)/71 (8.4)^***^	178 (69.5)/28 (10.9)	< 0.001
Consolidation [bilateral/unilateral]		1093 (33.4)/270 (8.3)	719 (32.9)/174 (8.0)	291 (34.8)/70 (8.4)	83 (32.8)/26 (10.3)	0.601
Treatment						
Remdesivir		1414 (39.3)	897 (36.8)	427 (47.7)^***^	90 (33.7)^###^	< 0.001
Molnupiravir		29 (6.0)	15 (5.3)	7 (5.1)	7 (11.5)	0.160
Corticosteroid		1838 (50.8)	1099 (44.9)	579 (64.1)^***^	160 (59.3)^***^	< 0.001
Tocilizumab		377 (10.5)	211 (8.6)	126 (14.1)^***^	40 (14.9)^***^	< 0.001
Baricitinib		252 (6.9)	165 (6.7)	80 (8.8)	7 (2.6)^**,###^	0.001

The data are presented as N (%) or median (interquartile range). The *P* value was calculated when comparing among the three groups

Abbreviations: BMI, body mass index; CKD, chronic kidney disease; COPD, chronic obstructive pulmonary disease; CRP, C-reactive protein; CT, computed tomography; eGFR, estimated glomerular filtration rate; GGO, ground glass opacity; HbA1c, hemoglobin a1c; KL-6, Krebs von den Lungen-6; LDH, lactate dehydrogenase; PCT, procalcitonin; UA, uric acid; WBC, white blood cell

* *P* < 0.0167, ** *P* < 0.01, *** *P* < 0.001: significant compared with Group 1

^#^*P* < 0.0167, ^##^*P* < 0.01, ^###^*P* < 0.001: significant compared with Group 2

a) Immunosuppressive therapy included corticosteroids (any dose), immunosuppressants, and biological agents

### Laboratory test results and imaging findings

Table 1 also shows a comparison of the laboratory results, imaging findings, and treatment among the three groups. Compared with patients in Group 1, those in Groups 2 and 3 had greater inflammatory responses (including higher white blood counts, C-reactive protein, procalcitonin, and D-dimer levels), decreased lymphocyte counts, and decreased albumin levels. Additionally, the patients in Group 3 had higher uric acid levels, lower lymphocyte counts, and lower albumin levels than those in Group 2. Patients in Group 2 had a higher incidence of bilateral ground glass opacities, consolidation, and rapid shadow spread on chest radiographs and computed tomography than those in Group 1, and patients in Group 3 had a higher incidence of rapid shadow spread than those in Group 1.

### Complications during hospitalization

**Fig. 1:**
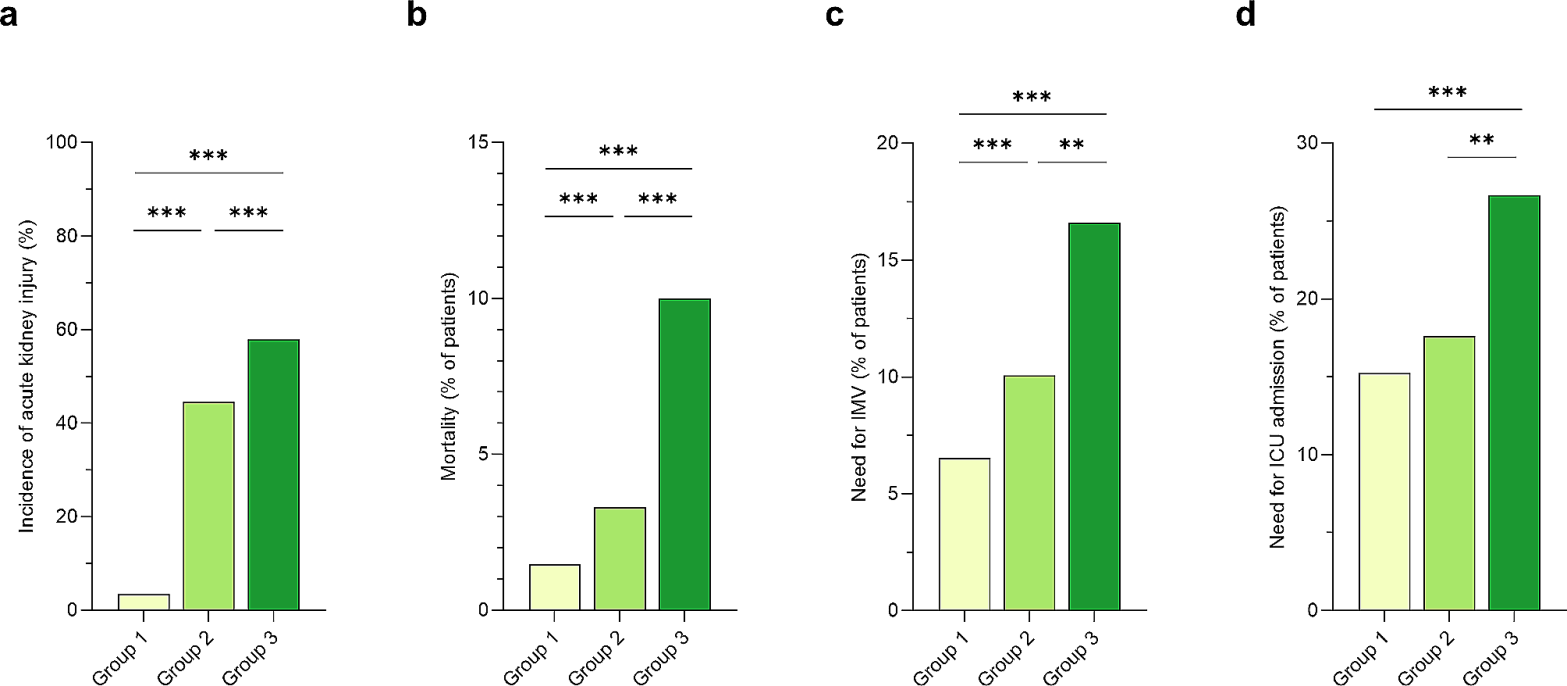
Acute kidney injury incidence and clinical outcomes in COVID-19 according to patient renal function status. (**a**) acute kidney injury; (**b**) mortality; (**c**) IMV requirement; (**d**) ICU admission. Statistical analysis used the chi-square test followed by Bonferroni adjustment. **P < 0.01, ***P < 0.001. Group 1 (*n* = 2,460), normal renal function on referral without a history of CKD; Group 2 (*n* = 905), impaired renal function on referral without a history of CKD; and Group 3 (*n* = 272), CKD history. CKD, chronic kidney disease; COVID-19, coronavirus disease; ICU, intensive care unit; IMV, invasive mechanical ventilation

Figure 1 and Additional file Fig. S3 show a comparison of complications of COVID-19 according to group. The incidence of bacterial infection and heart failure was higher in Groups 2 and 3 than in Group 1, and the incidence of heart failure was higher in Group 3 than in Group 2 (Additional file Fig. S3a, b). The incidence of thromboembolism was higher in Group 2 than in Group 1 (Additional file Fig. S3c). The incidence of AKI was higher in Groups 2 and 3 than in Group 1 (Fig. 1a).

Additional file Table S1 shows a comparison of clinical characteristics between patients with and without AKI. Patients in the AKI group were older, were more likely to be male, had higher BMI, were more likely to have a history of smoking, and were more likely to have a medical history of risk factors for renal impairment, including hypertension, diabetes mellitus, cardiovascular disease, and hyperuricemia, than those in the non-AKI group. The AKI group had a higher incidence of critical outcomes and higher rates of intensive care unit (ICU) admission, IMV support, and mortality than the non-AKI group.

### Clinical outcomes

**Fig. 2:**
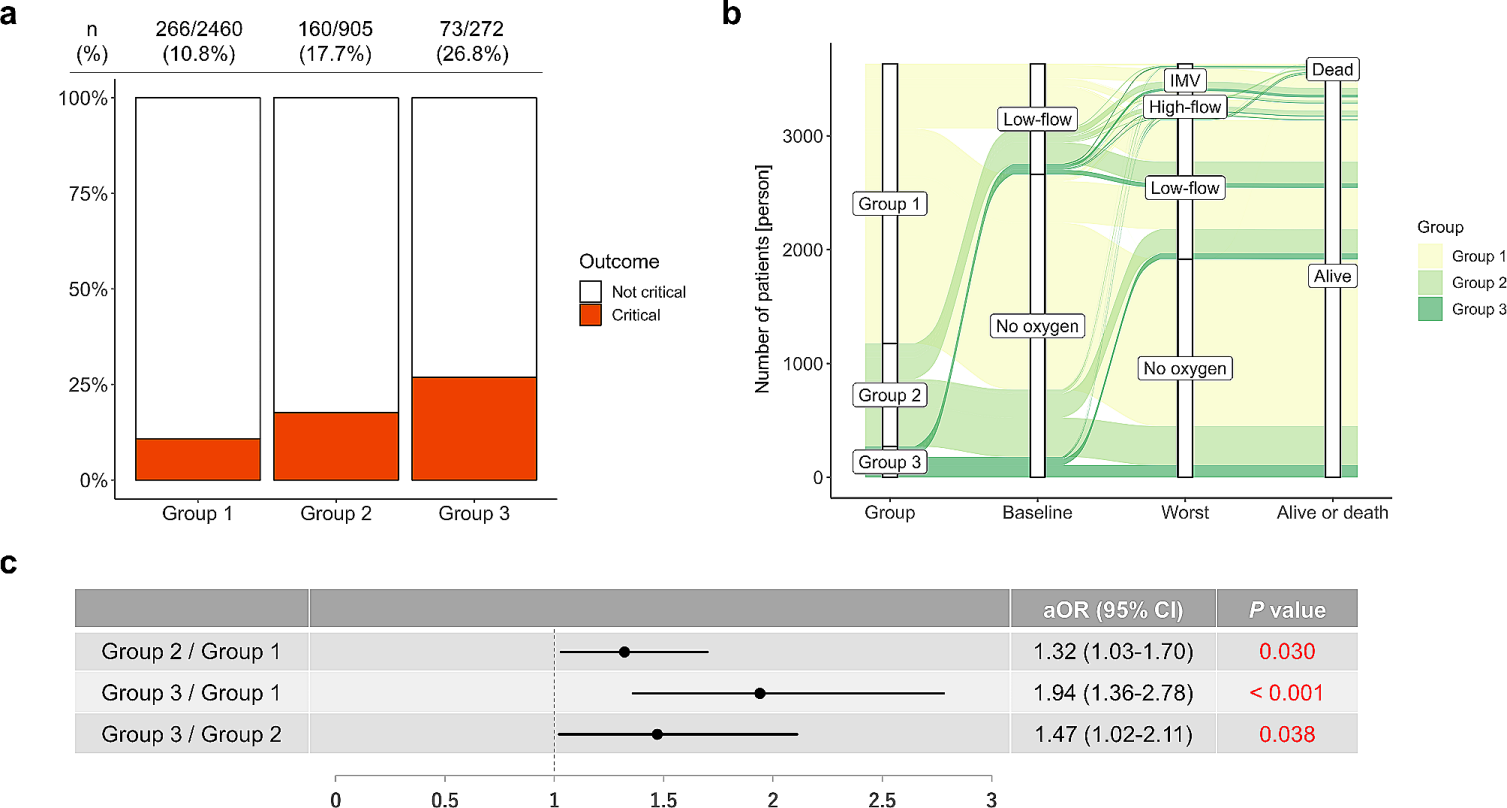
Incidence of critical outcomes of COVID-19 according to renal function status. (**a**) Incidence of critical outcomes by group. (**b**) Requirement for oxygen support on referral and at the worst point during COVID-19, and survival status. (**c**) Multivariable logistic regression analysis of the relationship between critical COVID-19 outcomes and renal function status, adjusted for age (</≥ 65 years); sex; BMI (</≥ 25 kg/m^2^); smoking history; and medical history of hypertension, diabetes mellitus, and cardiovascular disease. aOR, adjusted odds ratio; BMI, body mass index; CI, confidence interval; IMV, invasive mechanical ventilation

**Fig. 3:**
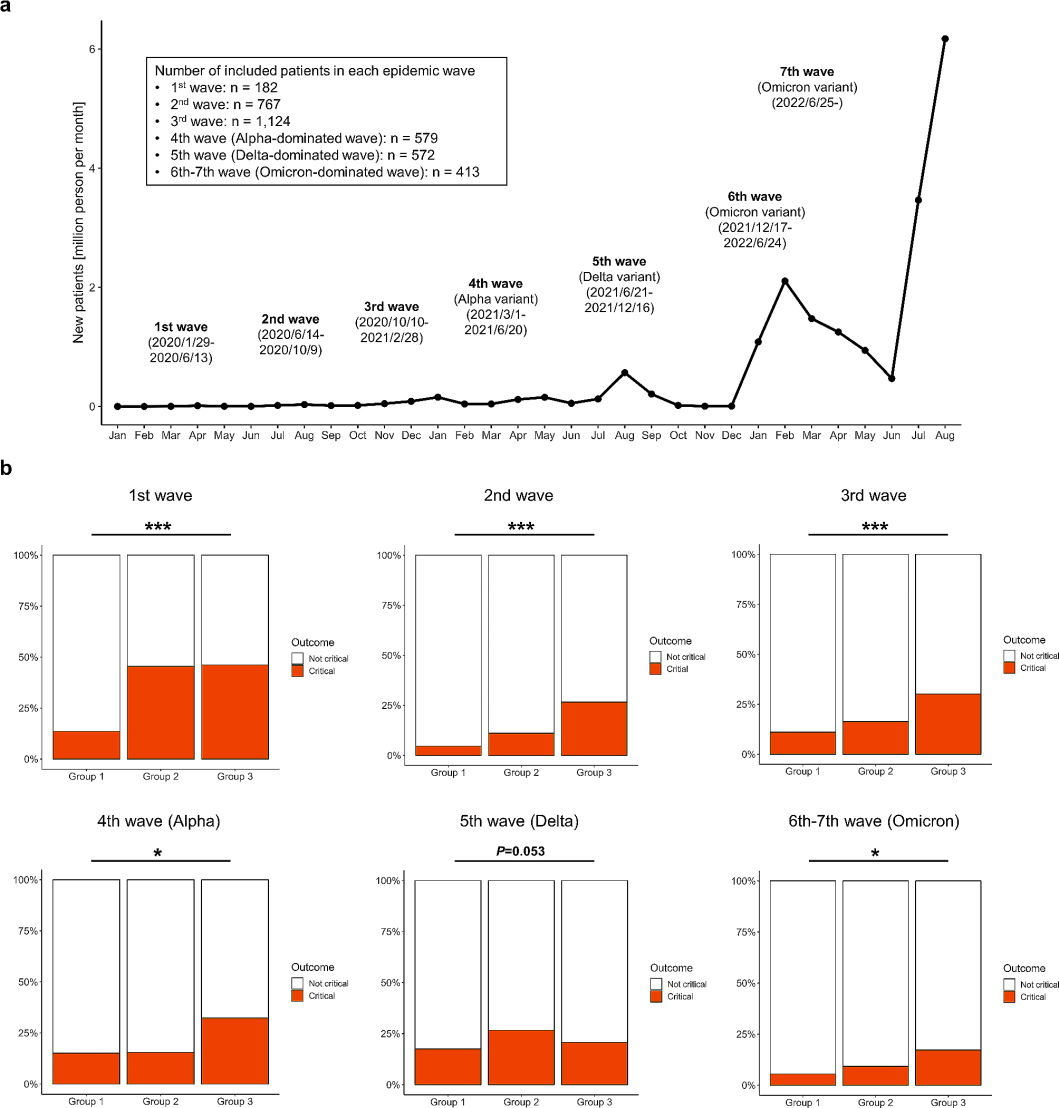
Comparison of the rates of critical outcomes among the patient groups according to the renal function status in each epidemic wave in Japan. (**a**) number of newly diagnosed COVID-19 patients and epidemic waves since January 2020 in Japan. (**b**) Comparison of the rates of critical outcomes in each epidemic wave. 1st wave, 2020/6/14-2020/10/9; 3rd wave, 2020-10/10-2021/2/28; 4th wave, Alpha variant-dominated wave, 2021/3/1-2021/6/20; 5th wave, Delta variant-dominated wave, 2021/6/21-2021/12/16; 6th and 7th wave, Omicron variant-dominated wave, 2021/12/17-2022/8/31. The *P* value was calculated when comparing among the three groups. ^*^P < 0.05, ^***^P < 0.001

The incidence of critical outcomes was 10.8% (266/2460) in Group 1, 17.7% (160/905) in Group 2, and 26.8% (73/272) in Group 3 (Fig. 2a). The mortality rate and the rate of requiring IMV support was lowest in Group 1 and highest in Group 3 (Fig. 1b, c). The rate of ICU admission was higher in Group 3 than in Groups 1 and 2 (Fig. 1d). A higher proportion of patients in Groups 2 and 3 than in Group 1 who were not on oxygen on referral required oxygen support during hospitalization (Fig. 2b). The incidence of needing IMV support, and the mortality rate, were highest in Group 3. Thirty-one (11.4%) patients on dialysis and five (1.8%) kidney transplant recipients were included in Group 3 (Table 1), and the stratification of these patient results did not reveal outcome differences in Group 3 (Additional file Fig. S4). Multivariable analysis, adjusted for previously reported risk factors, revealed that Groups 2 and 3 had an increased risk of critical outcomes compared with Group 1 (Group 2, adjusted odds ratio [aOR]: 1.32, 95% confidence interval [CI]: 1.03–1.70, *P* = 0.030; Group 3, aOR: 1.94, 95% CI: 1.36–2.78, *P* < 0.001), and the risk of critical outcomes was higher in Group 3 compared with Group 2 (aOR: 1.47, 95% CI: 1.02–2.11, *P* = 0.038) (Fig. 2c). Group 3 also had an increased risk of requiring IMV (aOR: 1.68, 95% CI: 1.09–2.59, *P* = 0.019) and death (aOR: 3.86, 95% CI: 1.97–7.56, *P* < 0.001) (Additional file Fig. S5). An analysis of the critical outcomes by group stratified by the times of epidemic waves showed similar results (Fig. 3), and univariate analysis also showed an increased risk of critical outcomes in Groups 2 and 3 compared with Group 1 (Additional file Table S2). Additional file Table S3 shows the comparisons of the critical outcomes based on the treatment for COVID-19 among the three groups according to renal function status. Regarding antiviral drugs, use of remdesivir was higher in Group 2, and the rate of requiring IMV support was higher in Group 3. Regarding treatment with immunosuppressants, Group 3 exhibited significantly poorer outcomes to corticosteroids and tocilizumab whereas Group 2 demonstrated outcomes comparable to those of Group 1.

The results of the univariate analyses for associations between eGFR, patient characteristics, and critical COVID-19 outcomes are shown in Table 2. In Group 1, older age, male sex, higher BMI, history of smoking, hypertension, diabetes, and AKI were significant predictors of critical outcomes. In Group 2, male sex (OR: 1.54, 95% CI: 1.05–2.26, *P* = 0.029), AKI (OR: 2.09, 95% CI: 1.46–2.98, *P* < 0.001), and eGFR < 30 mL/min/1.73 m^2^ (OR: 2.89, 95% CI: 1.64–4.98, *P* < 0.001) were significant predictors of critical outcomes. In Group 3, only eGFR < 30 mL/min/1.73 m^2^ (OR: 1.87, 95% CI: 1.08–3.23, *P* = 0.025) was a significant predictor of critical outcomes.

**Table 2:** Univariate logistic regression analysis of critical outcomes in each patient group

Variables	Group 1		Group 2		Group 3	
	OR (95% CI)	*P*-value	OR (95% CI)	*P*-value	OR (95% CI)	*P*-value
eGFR < 30 [mL/min/1.73 m^2^]			2.89 (1.64–4.98)	< 0.001	1.87 (1.08–3.23)	0.025
AKI during the COVID-19 course	3.17 (1.91–5.26)	< 0.001	2.09 (1.46–2.98)	< 0.001	1.53 (0.87–2.68)	0.141
Age ≥ 65 [years]	2.02 (1.55–2.62)	< 0.001	1.02 (0.72–1.45)	0.892	1.56 (0.87–2.80)	0.132
Sex, male	1.71 (1.27–2.28)	< 0.001	1.54 (1.05–2.26)	0.029	1.11 (0.59–2.08)	0.755
BMI ≥ 25 [kg/m^2^]	1.43 (1.10–1.85)	0.008	1.12 (0.78–1.60)	0.540	0.72 (0.40–1.28)	0.258
Smoking history	1.41 (1.08–1.83)	0.012	0.89 (0.62–1.28)	0.539	1.04 (0.60–1.82)	0.882
Hypertension	2.11 (1.61–2.77)	< 0.001	0.95 (0.68–1.34)	0.781	1.05 (0.57–1.94)	0.867
Diabetes mellitus	2.52 (1.89–3.37)	< 0.001	1.05 (0.71–1.55)	0.815	1.59 (0.92–2.73)	0.096
Cardiovascular disease	1.21 (0.74–1.97)	0.449	0.88 (0.54–1.41)	0.585	1.32 (0.76–2.27)	0.320

Group 1: normal renal function (eGFR ≥ 60 mL/min/1.73 m^2^) without CKD; Group 2: impaired renal function (eGFR < 60 mL/min/1.73 m^2^) without CKD; Group 3: CKD history. Abbreviations: AKI, acute kidney injury; BMI, body mass index; CI, confidence interval; CKD, chronic kidney disease; COVID-19, coronavirus disease 2019; eGFR, estimated glomerular filtration rate; OR, odds ratio

## Discussion

To our knowledge, this is the first study to compare COVID-19 severity and outcomes in patients with previously diagnosed CKD and those with impaired renal function without a CKD history. The analysis revealed that patients with impaired renal function on referral had a higher risk of critical outcomes and complications, even if they did not have a history of CKD. Furthermore, patients with a history of CKD had a higher incidence of critical outcomes than those with renal dysfunction but no history of CKD on referral. There is a lack of evidence on the effectiveness of treatment in patients with impaired renal function, making them an important target group for new therapeutic interventions.

The study is the largest study of Japanese patients with COVID-19 and impaired renal function reported to date and provides data regarding the status of CKD, renal function, and AKI in patients with COVID-19 in Japan. The prevalence of CKD and the incidence of AKI were 7.5% and 17.9%, respectively, which are similar to those reported in other countries [7, 17, 18].

In this study, patients with a history of CKD on referral had the highest number of critical outcomes among the three groups. Previous studies have reported poor COVID-19 outcomes, including increased mortality, in patients with pre-existing CKD [7, 19]. Ethnic differences in the clinical characteristics of COVID-19 [1, 20] and the incidence of CKD have been reported [21]. These results show that a history of CKD is an important predictor of poor outcomes in Japanese patients with COVID-19. Of the patients with CKD in our study, those who had an eGFR < 30 mL/min/1.73 m^2^ had a higher risk of critical outcomes than those with an eGFR of 30–60 mL/min/1.73 m^2^. This result is consistent with those of studies from other countries [1, 22–24]. Patients on dialysis have a higher risk of severe disease than patients with CKD who are not on dialysis [25], and continuous renal replacement therapy (CRRT) has been reported to be associated with COVID-19 severity [26], the small number of patients who received CRRT in this study makes it difficult to draw conclusions.

In this study, impaired renal function on referral, without a CKD history, was a risk factor for critical COVID-19 outcomes, although the risk was lower than that in patients with a history of CKD. In addition, the group with impaired renal function on referral, without a history of CKD, showed better response of anti-COVID-19 treatment than the group with CKD. Few reports have discussed the relationship between renal function on referral and poor outcomes, regardless of CKD history [19], and to our knowledge, no previous studies have compared COVID-19 outcomes in patients with impaired renal function with and without a history of CKD. Patients with impaired renal function on referral are likely to include both patients with undiagnosed CKD and those without CKD but with an AKI caused by COVID-19. Although we were unable to distinguish between the two in this study, the results suggest that the degree of renal impairment on referral may be an important biomarker for predicting poor outcomes.

Patients in Groups 2 and 3 in this study were older, more likely to be male, and had a higher prevalence of diabetes and other systemic comorbidities than those in Group 1. Many previous studies have reported that age; sex; and comorbidities, such as diabetes, hyperuricemia, and hypertension are associated with more severe COVID-19 [3, 4, 27, 28]. These characteristics may explain the higher incidence of critical outcomes in Groups 2 and 3. Patients in Groups 2 and 3 also had more pronounced inflammatory responses and lower lymphocyte counts than those in Group 1, consistent with previous reports that CKD is characterized by hyperinflammation and low lymphocyte counts [29, 30]. The finding that Groups 2 and 3 had a lower incidence of upper respiratory tract symptoms and higher incidence of lower respiratory tract symptoms, radiographic and computed tomographic shadows, and rapid deterioration, which are all poor prognostic factors, is also consistent with previous studies [5]. Among patients with severe or critical illness in Groups 2 and 3, 86.8% were treated with systemic corticosteroids, and 56.8% were treated with remdesivir. Patients with severe renal dysfunction (stage 4 severe CKD or requiring dialysis) were excluded from the clinical trial for remdesivir [31], and its safety in patients with CKD has not been established. Additional studies are therefore required to address the safety and effectiveness of antiviral drugs in patients with COVID-19 and renal failure.

In this study, patients with AKI had poorer clinical outcomes, consistent with previous findings [6, 9]. COVID-19 can cause AKI via direct viral tubular and glomerular damage, the effects of cytokine storms, hemodynamic effects, and drug side effects [32]. Notably, in this study, not only patients with a history of CKD but also patients with impaired renal function, on referral, without a CKD history, had an increased risk of developing AKI.

This study has some limitations. First, laboratory data prior to the COVID-19 diagnosis were unavailable, so we were not able to define CKD based on the Kidney Disease Improving Global Outcomes (KDIGO) guideline. The lack of previous laboratory data related to CKD is a major limitation. In this study, baseline renal function was calculated from laboratory data within 48 h of referral without renal imaging, and the history of CKD was based on patient interviews. Patients with impaired renal function on referral are likely to have included both patients with undiagnosed CKD and patients without CKD but with AKI caused by COVID-19. This could have introduced bias into the results. Although it was not possible to differentiate between these two groups due to the study design, we were able to define a group of patients with impaired renal function and no history of CKD on referral, thus identifying a new population of interest. Second, the absence of information on urinary findings may have resulted in an underestimation of the prevalence of CKD and an inability to detect mild renal dysfunction not manifesting as AKI. Because urinary findings and longitudinal laboratory data were not available, the diagnosis of AKI complications was defined by physician judgment based on the KDIGO guideline. In Japan, patients with COVID-19 were referred to hospitals with available beds according to government policy, rather than to their regular healthcare providers. Therefore, conducting follow-up assessments at the same hospital after discharge was often impractical, making it difficult to obtain pre- and post-discharge data. Further studies that include the laboratory findings during the disease course are needed. Third, this study did not assess the SARS-CoV-2 variant or collect complete information on patient vaccination status. However, we have already confirmed the stratified analysis based on the epidemic waves in Japan as reported [33, 34], and an analysis stratified by these epidemic waves corresponded to the viral variants in this study, confirming the robustness of the results. Future studies should consider stratifying the analysis according to the epidemics wave or viral variants.

## Conclusions

Patients without a CKD history but with impaired renal function on referral had poor clinical outcomes, and a CKD history further increased the risk. Clinicians should consider the presence of CKD and renal function at the time of COVID-19 diagnosis in the care and management of patients with COVID-19.

### Electronic supplementary material

Below is the link to the electronic supplementary material.

Supplementary Material 1

## Data Availability

The datasets used and/or analyzed during the current study are available from the corresponding author on reasonable request.

## Abbreviations

AKI: Acute kidney injury
BMI: Body mass index
CKD: Chronic kidney disease
COVID-19: Coronavirus disease
CRRT: Continuous renal replacement therapy
eGFR: Estimated glomerular filtration rate
IMV: Invasive mechanical ventilation

## References

[1] Williamson EJ, Walker AJ, Bhaskaran K (2020). Factors associated with COVID-19-related death using OpenSAFELY. Nature.

[2] Lee H, Chubachi S, Namkoong H (2022). Effects of mild obesity on outcomes in Japanese patients with COVID-19: a nationwide consortium to investigate COVID-19 host genetics. Nutr Diabetes.

[3] Fukushima T, Chubachi S, Namkoong H et al. Clinical significance of prediabetes, undiagnosed diabetes and diagnosed diabetes on critical outcomes in COVID-19: integrative analysis from the Japan COVID-19 task force. Diabetes Obes Metab. 2022. 10.1111/dom.14857 PMC9538969 36056760

[4] Fukushima T, Chubachi S, Namkoong H (2022). U-shaped association between abnormal serum uric acid levels and COVID-19 severity: reports from the Japan COVID-19 Task Force. Int J Infect Dis.

[5] Nakagawara K, Chubachi S, Namkoong H (2022). Impact of upper and lower respiratory symptoms on COVID-19 outcomes: a multicenter retrospective cohort study. Respir Res.

[6] Ng JH, Hirsch JS, Hazzan A (2021). Outcomes among patients hospitalized with COVID-19 and acute kidney Injury. Am J Kidney Dis.

[7] Jdiaa SS, Mansour R, El Alayli A (2022). COVID-19 and chronic kidney disease: an updated overview of reviews. J Nephrol.

[8] Kikuchi K, Nangaku M, Ryuzaki M (2021). Survival and predictive factors in dialysis patients with COVID-19 in Japan: a nationwide cohort study. Ren Replace Ther.

[9] Chan L, Chaudhary K, Saha A (2021). AKI in Hospitalized patients with COVID-19. J Am Soc Nephrol.

[10] Sato R, Matsuzawa Y, Ogawa H (2022). Chronic kidney disease and clinical outcomes in patients with COVID-19 in Japan. Clin Exp Nephrol.

[11] Tanaka H, Lee H, Morita A (2021). Clinical characteristics of patients with Coronavirus Disease (COVID-19): preliminary Baseline Report of Japan COVID-19 Task Force, a Nationwide Consortium To Investigate Host Genetics of COVID-19. Int J Infect Dis.

[12] Namkoong H, Edahiro R, Takano T (2022). DOCK2 is involved in the host genetics and biology of severe COVID-19. Nature.

[13] Matsuo S, Imai E, Horio M (2009). Revised equations for estimated GFR from serum creatinine in Japan. Am J Kidney Dis.

[14] Stefan N, Birkenfeld AL, Schulze MB (2021). Global pandemics interconnected - obesity, impaired metabolic health and COVID-19. Nat Rev Endocrinol.

[15] Petrilli CM, Jones SA, Yang J (2020). Factors associated with hospital admission and critical illness among 5279 people with coronavirus disease 2019 in New York City: prospective cohort study. BMJ.

[16] Stevens PE, Levin A, Kidney Disease: Improving Global Outcomes Chronic Kidney Disease Guideline Development Work Group M (2013). Evaluation and management of chronic kidney disease: synopsis of the kidney disease: improving global outcomes 2012 clinical practice guideline. Ann Intern Med.

[17] Singh J, Malik P, Patel N (2022). Kidney disease and COVID-19 disease severity-systematic review and meta-analysis. Clin Exp Med.

[18] Cheng Y, Luo R, Wang K (2020). Kidney disease is associated with in-hospital death of patients with COVID-19. Kidney Int.

[19] Portoles J, Marques M, Lopez-Sanchez P (2020). Chronic kidney disease and acute kidney injury in the COVID-19 Spanish outbreak. Nephrol Dial Transpl.

[20] Mackey K, Ayers CK, Kondo KK (2021). Racial and ethnic disparities in COVID-19-Related infections, hospitalizations, and deaths: a systematic review. Ann Intern Med.

[21] Webster AC, Nagler EV, Morton RL (2017). Chronic Kidney Disease Lancet.

[22] Gansevoort RT, Hilbrands LB (2020). CKD is a key risk factor for COVID-19 mortality. Nat Rev Nephrol.

[23] Council E-E, Group EW (2021). Chronic kidney disease is a key risk factor for severe COVID-19: a call to action by the ERA-EDTA. Nephrol Dial Transpl.

[24] Uribarri A, Nunez-Gil IJ, Aparisi A (2020). Impact of renal function on admission in COVID-19 patients: an analysis of the international HOPE COVID-19 (Health Outcome Predictive evaluation for COVID 19) Registry. J Nephrol.

[25] Flythe JE, Assimon MM, Tugman MJ (2021). Characteristics and outcomes of individuals with pre-existing kidney disease and COVID-19 admitted to Intensive Care Units in the United States. Am J Kidney Dis.

[26] Liu YF, Zhang Z, Pan XL (2021). The chronic kidney disease and acute kidney injury involvement in COVID-19 pandemic: a systematic review and meta-analysis. PLoS ONE.

[27] Kabia AU, Li P, Jin Z (2022). The effects of hypertension on the prognosis of coronavirus disease 2019: a systematic review and meta-analysis on the interactions with age and antihypertensive treatment. J Hypertens.

[28] Grasselli G, Greco M, Zanella A (2020). Risk factors Associated with Mortality among patients with COVID-19 in Intensive Care Units in Lombardy, Italy. JAMA Intern Med.

[29] D’Marco L, Puchades MJ, Romero-Parra M (2020). Coronavirus disease 2019 in chronic kidney disease. Clin Kidney J.

[30] Syed-Ahmed M, Narayanan M (2019). Immune Dysfunction and risk of infection in chronic kidney disease. Adv Chronic Kidney Dis.

[31] Beigel JH, Tomashek KM, Dodd LE (2020). Remdesivir for the treatment of Covid-19 - final report. N Engl J Med.

[32] Diao B, Wang C, Wang R (2021). Human kidney is a target for novel severe acute respiratory syndrome coronavirus 2 infection. Nat Commun.

[33] Lee H, Chubachi S, Namkoong H (2022). Characteristics of hospitalized patients with COVID-19 during the first to fifth waves of infection: a report from the Japan COVID-19 Task Force. BMC Infect Dis.

[34] Tanaka H, Chubachi S, Asakura T (2023). Characteristics and clinical effectiveness of COVID-19 vaccination in hospitalized patients in Omicron-dominated epidemic wave - a nationwide study in Japan. Int J Infect Dis.

