# Supplementary material for: Prognostic significance of chronic kidney disease and impaired renal function in Japanese patients with COVID-19
Source: BMC Infect Dis. 2024 May 25;24:527. doi: 10.1186/s12879-024-09414-w (PMC11128123; doi:10.1186/s12879-024-09414-w)
Supplement: Supplementary file 1 — Supplementary Material 1 [file 12879_2024_9414_MOESM1_ESM.docx]

**Prognostic Significance of Chronic Kidney Disease and Impaired Renal**

**Function in Japanese Patients with COVID-19**

**BMC Infectious Diseases**

**Additional File**

Hiromu Tanaka^1^, Shotaro Chubachi^1,a,*^, Takanori Asakura^1,2,3,a,*^, Ho Namkoong^4^, Shuhei Azekawa^1^,

Shiro Otake^1^, Kensuke Nakagawara^1^, Takahiro Fukushima^1^, Ho Lee^1^, Mayuko Watase^1^,

Kaori Sakurai^1^, Tatsuya Kusumoto^1^, Katsunori Masaki^1^, Hirofumi Kamata^1^, Makoto Ishii^1,5^,

Naoki Hasegawa^4^, Yukinori Okada^6,7,8^, Ryuji Koike^9^, Yuko Kitagawa^10^, Akinori Kimura^11^,

Seiya Imoto^12^, Satoru Miyano^13^, Seishi Ogawa^14^, Takanori Kanai^15^, Koichi Fukunaga^1^

^1^ Division of Pulmonary Medicine, Department of Medicine, Keio University School of Medicine, Tokyo, Japan

^2^ Department of Clinical Medicine (Laboratory of Bioregulatory Medicine), Kitasato University School of Pharmacy, Tokyo, Japan

^3^ Department of Respiratory Medicine, Kitasato University, Kitasato Institute Hospital, Tokyo, Japan

^4^ Department of Infectious Diseases, Keio University School of Medicine, Tokyo, Japan

^5^ Department of Respiratory Medicine, Nagoya University Graduate School of Medicine, Nagoya, Japan

^6^ Department of Statistical Genetics, Osaka University Graduate School of Medicine, Suita, Japan

^7^ Department of Genome Informatics, Graduate School of Medicine, the University of Tokyo, Tokyo, Japan

^8^ Laboratory for Systems Genetics, RIKEN Center for Integrative Medical Sciences, Kanagawa, Japan

^9^ Health Science Research and Development Center (HeRD), Tokyo Medical and Dental University, Tokyo, Japan

^10^ Department of Surgery, Keio University School of Medicine, Tokyo, Japan

^11^ Institute of Research, Tokyo Medical and Dental University, Tokyo, Japan

^12^ Division of Health Medical Intelligence, Human Genome Center, the Institute of Medical Science, the University of Tokyo, Tokyo, Japan

^13^ M&D Data Science Center, Tokyo Medical and Dental University, Tokyo, Japan

^14^ Department of Pathology and Tumor Biology, Kyoto University, Kyoto, Japan

^15^ Division of Gastroenterology and Hepatology, Department of Internal Medicine, Keio University School of Medicine, Tokyo, Japan

^a^ These authors contributed equally to this study.

*Corresponding Authors:

Shotaro Chubachi

Division of Pulmonary Medicine, Department of Medicine, Keio University School of Medicine

35 Shinanomachi, Shinjuku, Tokyo 160-8582, Japan

Tel: +81-3-3353-1211

E-mail: [bachibachi472000@z6.keio.jp](mailto:bachibachi472000@z6.keio.jp)

Takanori Asakura

Division of Pulmonary Medicine, Department of Medicine, Keio University School of Medicine

35 Shinanomachi, Shinjuku, Tokyo 160-8582, Japan

Tel: +81-3-3353-1211

E-mail: [takanori.asakura@gmail.com](mailto:takanori.asakura@gmail.com)


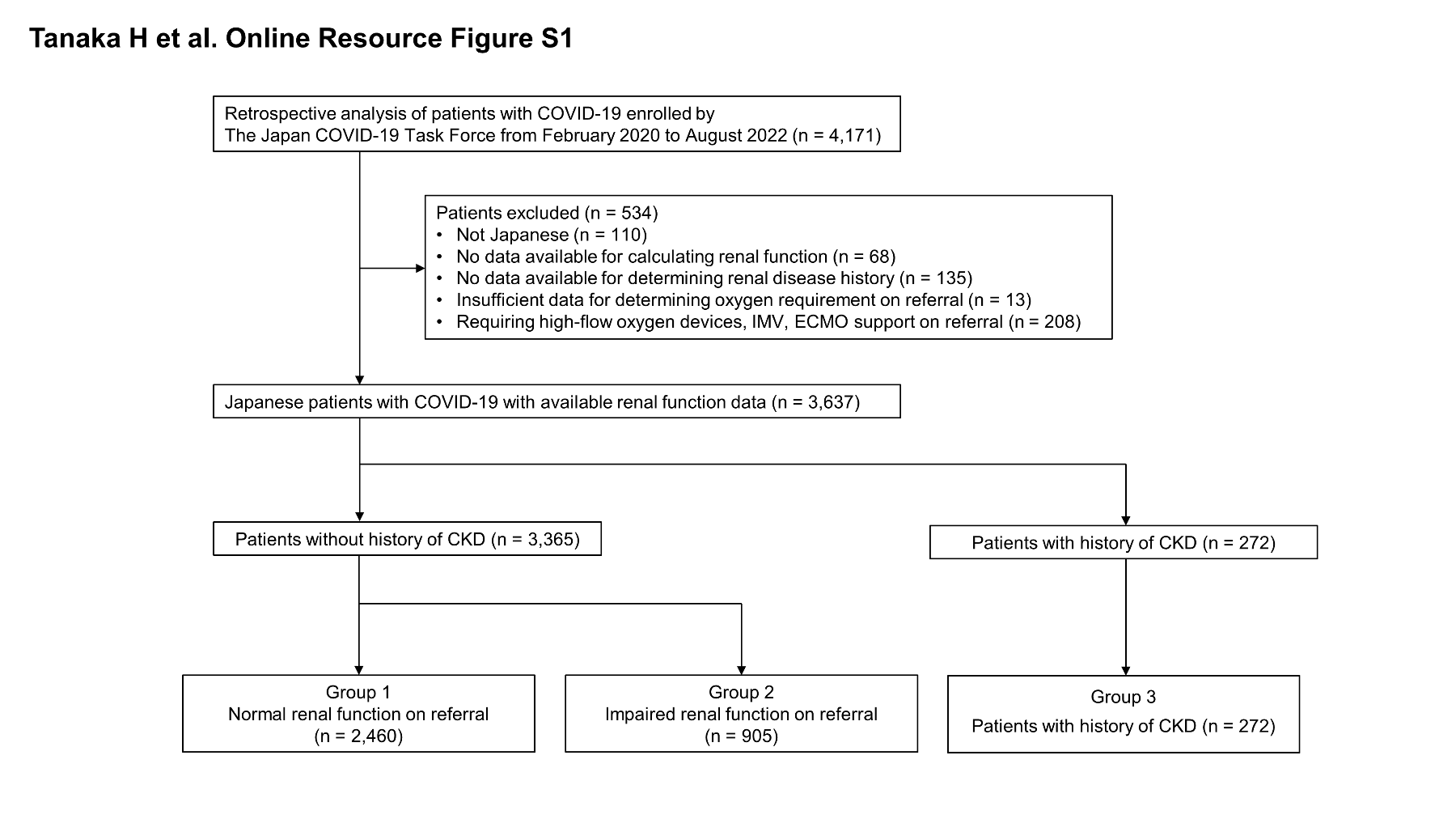


**Fig. S1** Consort diagram of patient selection. We classified the included patients into three groups: Group 1 (n=2,460), normal renal function (eGFR ≥ 60 ml/min/1.73 m^2^) on referral without CKD history; Group 2 (n=905), impaired renal function (eGFR < 60 ml/min/1.73 m^2^) on referral without CKD history; Group 3 (n=272), CKD history. CKD, chronic kidney disease; COVID-19, coronavirus disease 2019; ECMO, extracorporeal membrane oxygenation; eGFR, estimated glomerular filtration rate; IMV, invasive mechanical ventilation.

#
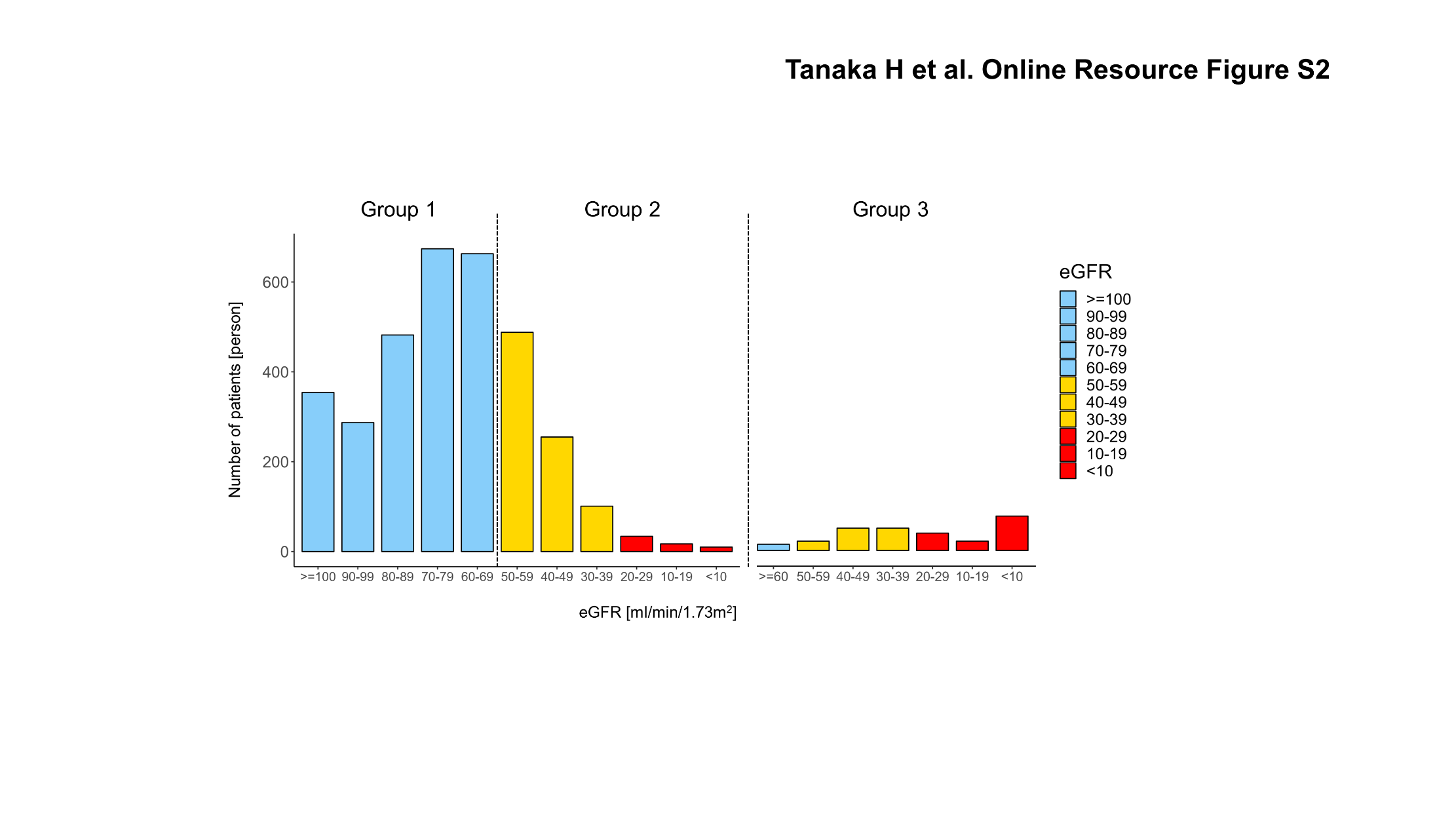


**Fig. S2** The distribution of eGFR (≥100, 90-99, 80-89, 70-79, 60-69, 50-59, 40-49, 30-39, 20-29, 10-19, and <10 mL/min/1.73 m^2^) in the included patients. The blue bar shows normal renal function on referral (i.e., eGFR ≥ 60 mL/min/1.73 m^2^). The yellow and red bars show impaired renal function on referral (i.e., yellow bar, eGFR < 60 mL/min/1.73 m^2^; red bar, eGFR < 30 mL/min/1.73 m^2^). eGFR, estimated glomerular filtration rate.


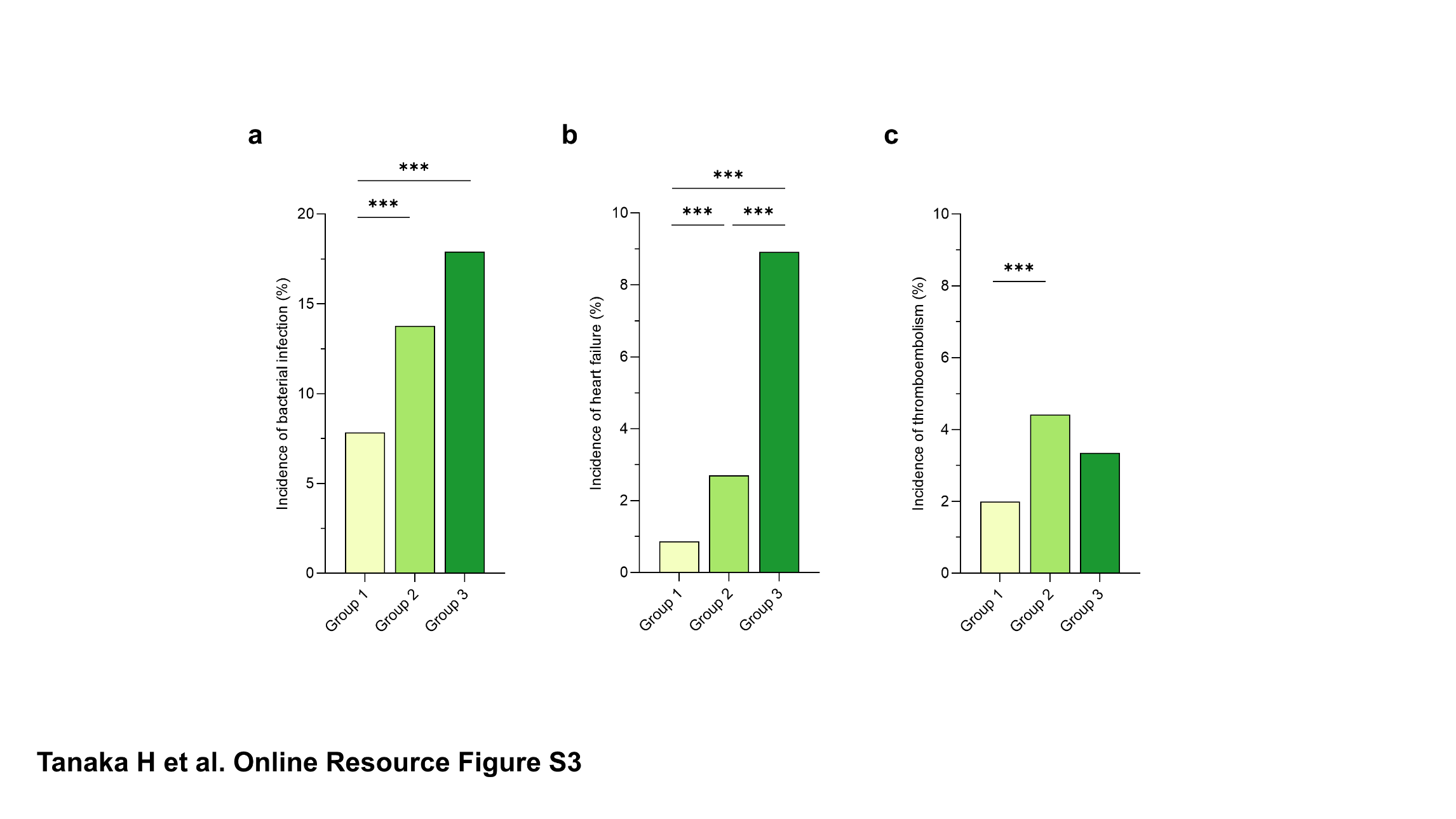


**Fig. S3** The incidence of post-hospital complications (**a**, bacterial infection; **b**, heart failure; **c**, thromboembolism) of COVID-19 among the patient groups. Statistical analysis was performed using the chi-square test followed by Bonferroni adjustment. ****P*<0.001. COVID-19, coronavirus disease 2019.


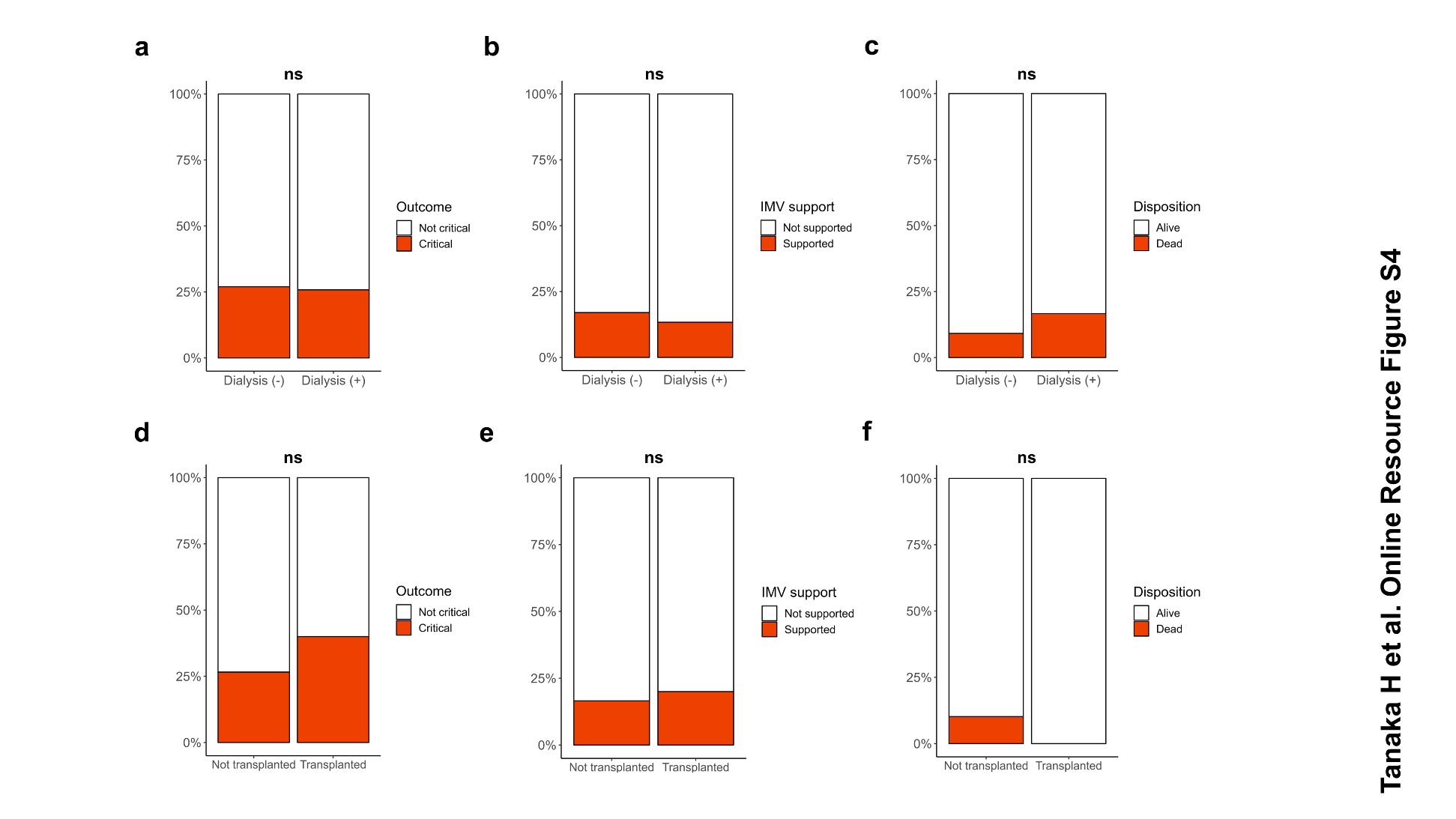


**Fig. S4** The comparison of the rates of clinical outcomes between patients with and without continuous renal replacement therapy. a-c, with or without dialysis; d-f, with or without kidney transplant. The clinical outcomes are as follows: a and d, critical outcomes; b and e, need for IMV support; c and f, death. IMV, invasive mechanical ventilation; ns, not significant.


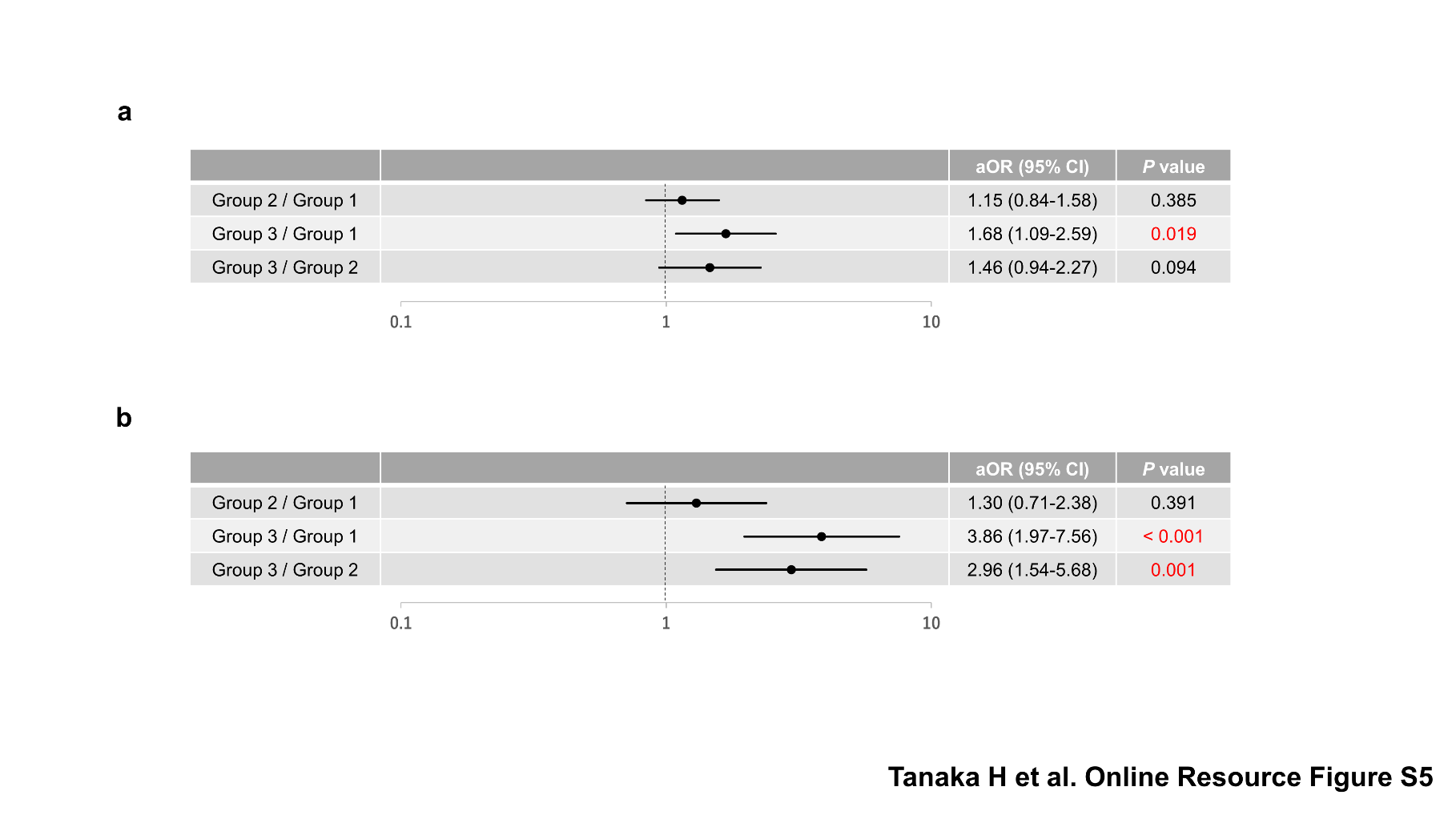


**Fig. S5** Multivariable analysis of the relationship between the clinical outcomes (a, need for IMV; b, death) and patient groups according to the renal function status, adjusted for age (</≥ 65 years); sex; BMI (</≥25 kg/m^2^); smoking history; and medical history of hypertension, diabetes mellitus, and cardiovascular disease. aOR, adjusted odds ratio; BMI, body mass index; CI, confidence interval; IMV, invasive mechanical ventilation.

**Table S1** Comparison of clinical background characteristics and disease outcomes between the non-AKI and AKI groups

| **Parameters** | **Normal ranges** | **Non-AKI group** | **AKI group** | ***P*-value** |
| --- | --- | --- | --- | --- |
|  |  | **(n=2,876)** | **(n=627)** |  |
| **Background variables** |  |  |  |  |
| Age [years] |  | 56 (43-70) | 68 (57-77) | <0.001 |
| Sex, male |  | 1867 (64.9) | 461 (73.5) | <0.001 |
| BMI [kg/m^2^] |  | 23.8 (21.3-26.9) | 24.6 (22.0-27.5) | 0.008 |
| Smoking history |  |  |  | 0.005 |
| Never |  | 1443 (53.7) | 282 (47.4) |  |
| Previously or currently |  | 1245 (45.9) | 313 (52.6) |  |
| Comorbidities |  |  |  |  |
| Hypertension |  | 849 (29.7) | 365 (58.3) | <0.001 |
| Diabetes mellitus |  | 526 (18.4) | 191 (30.8) | <0.001 |
| Cardiovascular disease |  | 268 (9.4) | 130 (20.9) | <0.001 |
| Malignancy |  | 223 (7.8) | 67 (10.8) | 0.016 |
| Autoimmune disease |  | 150 (5.3) | 31 (5.0) | 0.777 |
| COPD |  | 134 (4.7) | 33 (5.3) | 0.509 |
| Asthma |  | 204 (7.2) | 44 (7.1) | 0.921 |
| Hyperuricemia |  | 216 (7.6) | 135 (21.7) | <0.001 |
| Chronic liver disease |  | 123 (4.3) | 37 (5.9) | 0.077 |
| Immunosuppressive therapy^a)^ |  | 139 (4.9) | 33 (5.3) | 0.639 |
| Symptoms |  |  |  |  |
| Fever (≥37.5℃) |  | 2306 (80.9) | 522 (83.8) | 0.089 |
| Cough |  | 1835 (64.4) | 394 (64.2) | 0.919 |
| Sputum |  | 878 (31.0) | 197 (32.1) | 0.572 |
| Sore throat |  | 805 (28.5) | 139 (22.6) | 0.003 |
| Rhinorrhea |  | 420 (14.9) | 79 (13.0) | 0.232 |
| Dysgeusia |  | 533 (18.8) | 66 (10.8) | <0.001 |
| Dysosmia |  | 459 (16.2) | 47 (7.7) | <0.001 |
| Shortness of breath |  | 996 (35.4) | 286 (46.7) | <0.001 |
| Fatigue |  | 1510 (53.3) | 392 (63.4) | <0.001 |
| Laboratory data |  |  |  |  |
| WBC count [/μL] | 3300–8600 | 5000 (4000-6600) | 5600 (4200-7400) | <0.001 |
| Neutrophil [/μL] | 1800–7500 | 3440 (2490-4920) | 4090 (2910-5770) | <0.001 |
| Lymphocyte [/μL] | 1000–4800 | 990 (710-1350) | 820 (600-1170) | <0.001 |
| PCT [ng/mL] | <0.04 | 0.06 (0.04-0.10) | 0.13 (0.07-0.31) | <0.001 |
| CRP [mg/dL] | <0.14 | 2.74 (0.64-6.97) | 5.78 (2.50-11.06) | <0.001 |
| D-dimer [μg/mL] | <0.90 | 0.90 (0.50-1.38) | 1.12 (0.70-1.99) | <0.001 |
| **Outcomes** |  |  |  |  |
| Critical outcome |  | 322 (11.2) | 159 (25.4) | <0.001 |
| ICU admission |  | 424 (14.8) | 158 (25.3) | <0.001 |
| IMV support |  | 189 (6.6) | 93 (14.9) | <0.001 |
| Death |  | 46 (1.6) | 44 (7.0) | <0.001 |

The data are presented as N (%) or median (interquartile range).

Abbreviations: AKI, acute kidney injury; BMI, body mass index; COPD, chronic obstructive pulmonary disease; CRP, C-reactive protein; ICU, intensive care unit; IMV, invasive mechanical ventilation; PCT, procalcitonin; WBC, white blood cell.

a) Immunosuppressive therapy included corticosteroids (any dose), immunosuppressants, and biological agents.

| **Table S2**　Univariate logistic regression analysis of the relationship between critical outcomes and renal function status in each epidemic wave | | | | | | |
| --- | --- | --- | --- | --- | --- | --- |
| **Variables** | **1st wave (n=182)** | **2nd wave (n=767)** | **3rd wave (n=1,124)** | **4th wave (n=579)** | **5th wave (n=572)** | **6-7th waves (n=413)** |
| Group 2 / Group 1 | 5.29 (2.42-11.6)^***^ | 2.55 (1.33-4.87)^**^ | 1.57 (1.07-2.32)^*^ | 1.02 (0.61-1.72) | 1.71 (1.11-2.65)^*^ | 1.75 (0.76-4.03) |
| Group 3 / Group 1 | 5.45 (1.63-18.2)^**^ | 7.42 (3.45-16.0)^***^ | 3.47 (2.11-5.72)^***^ | 2.68 (1.24-5.78)^*^ | 1.23 (0.48-3.13) | 3.57 (1.48-8.63)^**^ |
| Group 3 / Group 2 | 1.03 (0.30-3.56) | 2.91 (1.26-6.74)^*^ | 2.21 (1.28-3.80)^**^ | 2.62 (1.13-6.10)^*^ | 0.72 (0.27-1.88) | 2.05 (0.81-5.14) |
| The data are presented as odds ratios (95% CI).  1st wave, 2020/1/29-2020/6/13; 2nd wave, 2020/6/14-2020/10/9; 3rd wave, 2020-10/10-2021/2/28; 4th wave, Alpha variant-dominated wave, 2021/3/1-2021/6/20; 5th wave, Delta variant-dominated wave, 2021/6/21-2021/12/16; 6th and 7th wave, Omicron variant-dominated wave, 2021/12/17-2022/8/31. Abbreviations: CI, confidence interval. ^*^ *P* <0.05, ^**^ *P* <0.01, ^***^ *P* <0.001. | | | | | | |

| **Table S3**　Comparison of the critical outcomes based on the treatment for COVID-19 among the three groups according to the renal function status | | | | | |
| --- | --- | --- | --- | --- | --- |
| **Parameters** | **All (n=3,637)** | **Group 1 (n=2,460)** | **Group 2 (n=905)** | **Group 3 (n=272)** | ***P* value** |
|  | All patients | History of CKD (-) Impaired renal function (-) | History of CKD (-) Impaired renal function (+) | History of CKD (+) |  |
| **Remdesivir** | **1414 (39.3)** | **897 (36.8)** | **427 (47.7)^***^** | **90 (33.7)^###^** | **<0.001** |
| Critical outcome | 331 (23.4) | 204 (22.7) | 99 (23.2) | 28 (31.1) | 0.201 |
| ICU admission | 317 (22.7) | 205 (23.2) | 86 (20.3) | 26 (28.9) | 0.179 |
| IMV support | 206 (14.6) | 127 (14.2) | 58 (13.6) | 21 (23.6) | 0.044 |
| Death | 44 (3.1) | 24 (2.7) | 16 (3.8) | 4 (4.4) | 0.441 |
| **Molnupiravir** | **29 (6.0)** | **15 (5.3)** | **7 (5.1)** | **7 (11.5)** | **0.160** |
| Critical outcome | 2 (6.9) | 0 (0.0) | 1 (14.3) | 1 (14.3) | 0.316 |
| ICU admission | 1 (3.5) | 0 (0.0) | 0 (0.0) | 1 (14.3) | 0.196 |
| IMV support | 0 (0.0) | 0 (0.0) | 0 (0.0) | 0 (0.0) |  |
| Death | 1 (3.5) | 0 (0.0) | 0 (0.0) | 1 (14.3) | 0.196 |
| **Corticosteroids** | **1838 (50.8)** | **1099 (44.9)** | **579 (64.1)^***^** | **160 (59.3)^***^** | **<0.001** |
| Critical outcome | 464 (25.2) | 249 (22.7) | 149 (25.7) | 66 (41.3)^***,###^ | <0.001 |
| ICU admission | 478 (26.3) | 277 (25.6) | 139 (24.3) | 62 (38.8)^***,###^ | <0.001 |
| IMV support | 270 (14.7) | 148 (13.5) | 82 (14.2) | 40 (25.0)^***,##^ | <0.001 |
| Death | 84 (4.6) | 32 (2.9) | 28 (4.8) | 24 (15.1)^***,###^ | <0.001 |
| **Tocilizumab** | **377 (10.5)** | **211 (8.6)** | **126 (14.1)^***^** | **40 (14.9)^***^** | **<0.001** |
| Critical outcome | 193 (51.2) | 108 (51.2) | 59 (46.8) | 26 (65.0) | 0.134 |
| ICU admission | 178 (47.6) | 103 (49.5) | 52 (41.3) | 23 (57.5) | 0.142 |
| IMV support | 126 (33.4) | 70 (33.2) | 36 (28.6) | 20 (50.0)^#^ | 0.043 |
| Death | 32 (8.5) | 11 (5.3) | 11 (8.7) | 10 (25.0)^***,##^ | <0.001 |
| **Baricitinib** | **252 (6.9)** | **165 (6.7)** | **80 (8.8)** | **7 (2.6)^**,###^** | **0.001** |
| Critical outcome | 79 (31.4) | 48 (29.1) | 28 (35.0) | 3 (42.9) | 0.518 |
| ICU admission | 61 (24.6) | 40 (24.7) | 20 (25.3) | 1 (14.3) | 0.809 |
| IMV support | 26 (10.4) | 13 (7.9) | 13 (16.3) | 0 (0.0) | 0.089 |
| Death | 6 (2.4) | 3 (1.8) | 2 (2.5) | 1 (14.3) | 0.106 |
| The data are presented as N (%). The *P* value was calculated when comparing among the three groups.  Abbreviations: CKD, chronic kidney disease; ICU, intensive care unit; IMV, invasive mechanical ventilation.  ^*^ *P*<0.0167, ^**^ *P*<0.01, ^***^ *P*<0.001: significant compared with Group 1. ^#^ *P*<0.0167, ^##^ *P*<0.01, ^###^ *P*<0.001: significant compared with Group 2. | | | | | |

**Acknowledgments**

We would like to thank all the participants involved in this study and all the members of the Japan COVID-19 Task Force who regularly engaged in clinical and research work on COVID-19. All the members contributed to this study. The list of members is shown below.

**Chiba University**

Koutaro Yokote, Taka-Aki Nakada, Ryuzo Abe, Taku Oshima, Tadanaga Shimada

**Daini Osaka Police Hospital**

Kensuke Kanaoka, Shoichi Ihara, Kiyoshi Komuta

**Eiju General Hospital**

Fumitake Saito, Keiko Mitamura, Masao Hagihara, Junichi Ochi, Tomoyuki Uchida

**Fujioka General Hospital**

Mitsuru Motegi

**Fujisawa City Hospital**

Masanori Nishikawa, Makoto Masuda, Aya Wakabayashi, Hiroki Watanabe, Suguru Ueda

**Fukujuji Hospital**

Takashi Yoshiyama, Ken Ohta, Hiroyuki Kokuto, Hideo Ogata, Yoshiaki Tanaka, Kenichi Arakawa, Masafumi Shimoda, Takeshi Osawa

**Fukuoka Tokushukai Hospital**

Nobuhiro Kodama, Yasunari Kaneyama, Shunsuke Maeda, Takashige Kuraki, Takemasa Matsumoto

**Fukuoka University Hospital**

Tohru Takata, Yoshihiko Nakamura, Kota Hoshino, Junichi Maruyama, Hiroyasu Ishikura

**Fukushima Medical University**

Yoko Shibata, Yoshinori Tanino, Takefumi Nikaido, Hiroyuki Minemura, Yuki Sato

**Gifu University**

Yuichiro Kitagawa, Tetsuya Fukuta, Takahito Miyake, Shozo Yoshida, Shinji Ogura

**Gunma University**

Masakiyo Yatomi, Toshitaka Maeno

**The Institute of Medical Science, The University of Tokyo**

Takayoshi Hyugaji, Eigo Shimizu, Kotoe Katayama, Seiya Imoto

**International University of Health and Welfare Shioya Hospital**

Akira Umeda, Kazuya Miyagawa, Hisato Shimada, Mayu Endo, Yoshiyuki Ohira

**Ishikawa Prefectural Central Hospital**

Koichi Nishi, Masaru Nishitsuji, Mayuko Tani, Junya Suzuki, Hiroki Nakatsumi

**JA Toride Medical Hospital**

Shinichi Ogawa, Tomouki Ogata, Shoichiro Ishihara

**Japan Community Health Care Organization Kanazawa Hospital**

Kazuyoshi Watanabe

**Japan Community Health Care Organization Saitama Medical Center**

Soichiro Ueda, Mamoru Sasaki, Ai Tada, Masayoshi Miyawaki, Masaomi Yamamoto, Eriko Yoshida, Reina Hayashi, Tomoki Nagasaka, Sawako Arai, Yutaro Kaneko, Kana Sasaki

**Japanese Red Cross Medical Center**

Takehiro Izumo, Minoru Inomata, Naoyuki Kuse, Nobuyasu Awano, Mari Tone

**Juntendo University**

Norihiro Harada, Masako Ichikawa, Kazuhisa Takahashi, Toshio Naito, Makoto Hiki, Yasushi Matsushita, Haruhi Takagi, Ryousuke Aoki, Ai Nakamura, Sonoko Harada, Hitoshi Sasano

**Kanagawa Cardiovascular and Respiratory Center**

Takashi Ogura, Hideya Kitamura, Eri Hagiwara, Kota Murohashi, Hiroko Okabayashi

**Kansai Electric Power Hospital**

Yuichiro Yamada, Takuya Hashino, Masato Shinoki

**Kansai Medical University General Medical Center**

Fukuki Saito, Yasushi Nakamori, Kazuhisa Yoshiya, Tomoyuki Yoshihara, Daiki Wada, Hiromu Iwamura, Syuji Kanayama, Shuhei Maruyama

**Kanto Rosai Hospital**

Yoshihiro Hirai, Hidetoshi Kawashima, Atsuya Narita, Kazuki Niwa, Yoshiyuki Sekikawa

**Kawasaki Municipal Ida Hospital**

Yasushi Nakano, Yukiko Nakajima, Ryusuke Anan, Ryosuke Arai, Yuko Kurihara, Yuko Harada, Kazumi Nishio

**Keio University**

Ho Namkoong, Shotaro Chubachi, Hiromu Tanaka, Yasunori Sato, Takanori Asakura, Tatsuya Kusumoto, Kaori Sakurai, Mayuko Watase, Takahiro Fukushima, Ho Lee, Shiro Otake, Kensuke Nakagawara, Yasushi Kondo, Yuko Kaneko, Hiroshi Nishihara, Yohei Mikami, Rino Ishihara, Yuta Matsubara, Junko Hamamoto, Emmy Yanagita, Mari Katsumata, Atsuho Morita, Takunori Ogawa, Hiroki Kabata, Katsunori Masaki, Hirofumi Kamata, Shinnosuke Ikemura, Satoshi Okamori, Hideki Terai, Junichi Sasaki, Hiroshi Morisaki, Yoshifumi Uwamino, Kosaku Nanki, Sho Uchida, Shunsuke Uno, Tomoyasu Nishimura, Toshiro Sato, Makoto Ishii, Naoki Hasegawa, Yuko Kitagawa, Takanori Kanai, Koichi Fukunaga

**Keiyu Hospital**

Tetsuya Shiomi, Kazuma Yagi, Mizuha Hashiguchi, Junko Kagyo

**KINSHUKAI Hanwa The Second Hospital**

Minoru Takada, Hidenori Kanda

**Kiryu Kosei General Hospital**

Mitsuyoshi Utsugi, Akihiro Ono

**Kitasato University**

Tomomi Takano, Kazuhiko Katayama

**Kitasato University Kitasato Institute Hospital**

Yusuke Suzuki, Sohei Nakayama, Keita Masuzawa

**KKR Sapporo Medical Center**

Satoshi Fuke, Hiroshi Saito

**Kobe University**

Shohei Makino, Moritoki Egi

**Kumamoto City Hospital**

Hajime Iwagoe, Hiroshi Takahashi, Kazuhiko Fujii, Hiroto Kishi

**Kyoto Prefectural University of Medicine**

Satoru Hashimoto, Masaki Yamasaki, Yu Kasamatsu

**Kyoto University**

Ryunosuke Saiki, Yasuhito Nannya, Seishi Ogawa

**Kyushu University**

Satoru Fukuyama, Yoshihiro Eriguchi, Akiko Yonekawa, Keiko Kan-o, Koichiro Matsumoto

**Matsumoto City Hospital**

Akihiro Ito

**Musashino Red Cross Hospital**

Namiki Izumi, Kaoru Nagata, Ken Ueda, Reiko Taki, Satoko Hanada

**Nagoya University**

Naozumi Hashimoto, Keiko Wakahara, Sakamoto Koji, Norihito Omote, Akira Ando

**National Center for Global Health and Medicine**

Yosuke Omae, Katsushi Tokunaga

**National Defense Medical College**

Yoshifumi Kimizuka, Akihiko Kawana, Tomoya Sano, Chie Watanabe, Ryohei Suematsu

**National Hospital Organization Hokkaido Medical Center**

Toshio Odani, Masaru Amishima, Takeshi Hattori, Yasuo Shichinohe

**National Hospital Organization Kanazawa Medical Center**

Takashi Kagaya, Toshiyuki Kita, Kazuhide Ohta, Satoru Sakagami, Kiyoshi Koshida

**National Hospital Organization Kumamoto Medical Center**

Masahiro Harada, Takeshi Takahashi, Hiroshi Ono, Toshihiro Sakurai, Takayuki Shibusawa

**National Hospital Organization Kyoto Medical Center**

Kei Nishiyama, Mariko Terashima, Satoru Beppu, Kosuke Yoshida

**National Hospital Organization Kyushu Medical Center**

Masaki Okamoto, Sayoko Ishihara, Masatoshi Shimo, Yoshihisa Tokunaga

**National Hospital Organization Saitama Hospital**

Shinichi Hayashi, Mai Takahashi, Mizuki Kuramochi, Isamu Kamimaki, Yoshiteru Tominaga

**National Hospital Organization Tokyo Hospital**

Osamu Narumoto, Hideaki Nagai, Nobuharu Ooshima

**National Hospital Organization Tokyo Medical Center**

Takao Mochimaru, Shigenari Nukaga, Ryosuke Satomi, Yoshitaka Oyamada, Nobuaki Mori

**Nihon University Itabashi Hospital**

Yasuhiro Gon, Kentaro Hayashi, Tetsuo Shimizu, Yutaka Kozu, Hisato Hiranuma

**Nippon Medical School**

Toru Tanaka, Takeru Kashiwada, Kazue Fujita, Yoshinobu Saito, Masahiro Seike

**Okayama Rosai Hospital**

Arihiko Kanehiro, Shinji Ozaki, Yasuko Fuchimoto, Sae Wada, Nobukazu Fujimoto

**Ome Municipal General Hospital**

Yu Kusaka, Takehiko Ohba, Susumu Isogai, Aki Ogawa, Takuya Inoue

**Osaka Saiseikai Nakatsu Hospital**

Tetsuya Ueda, Masanori Azuma, Ryuichi Saito, Toshikatsu Sado, Yoshimune Miyazaki, Ryuichi Sato, Yuki Haruta, Tadao Nagasaki, Yoshinori Yasui, Yoshinori Hasegawa

**Osaka University**

Ryuya Edahiro, Yuya Shirai, Kyuto Sonehara, Daisuke Okuzaki, Daisuke Motooka, Masahiro Kanai, Tatsuhiko Naito, Kenichi Yamamoto, Qingbo S Wang, Yasuhiro Kato, Takayoshi Morita, Shinichi Namba, Ken Suzuki, Yoko Naito, Yu-Chen Liu, Ayako Takuwa, Fuminori Sugihara, James B Wing, Shuhei Sakakibara, Nobuyuki Hizawa, Takayuki Shiroyama, Satoru Miyawaki, Yusuke Kawamura, Akiyoshi Nakayama, Hirotaka Matsuo, Yuichi Maeda, Takuro Nii, Yoshimi Noda, Takayuki Niitsu, Yuichi Adachi, Takatoshi Enomoto, Saori Amiya, Reina Hara, Yuta Yamaguchi, Teruaki Murakami, Tomoki Kuge, Kinnosuke Matsumoto, Yuji Yamamoto, Makoto Yamamoto, Midori Yoneda, Toshihiro Kishikawa, Shuhei Yamada, Shuhei Kawabata, Noriyuki Kijima, Masatoshi Takagaki, Noah Sasa, Yuya Ueno, Motoyuki Suzuki, Norihiko Takemoto, Hirotaka Eguchi, Takahito Fukusumi, Takao Imai, Munehisa Fukushima, Haruhiko Kishima, Hidenori Inohara, Kazunori Tomono, Kazuto Kato, Meiko Takahashi, Fumihiko Matsuda, Haruhiko Hirata, Yoshito Takeda, Atsushi Kumanogoh, Yukinori Okada

**St. Marianna University School of Medicine**

Tomoya Tsuchida, Shigeki Fujitani, Mumon Takita, Daiki Morikawa, Toru Yoshida

**St. Marianna University School of Medicine, Yokohama City Seibu Hospital**

Yuko Komase, Naoya Hida, Takahiro Tsuburai, Baku Oyama

**Saiseikai Kumamoto Hospital**

Kodai Kawamura, Kazuya Ichikado, Kenta Nishiyama, Hiroyuki Muranaka, Kazunori Nakamura

**Saiseikai Utsunomiya Hospital**

Ichiro Nakachi, Rie Baba, Daisuke Arai, Takayuki Ogura, Hidenori Takahashi, Shigehiro Hagiwara, Genta Nagao, Shunichiro Konishi

**Saiseikai Yokohamashi Nanbu Hospital**

Naoki Miyazawa, Yasuhiro Kimura, Reiko Sado, Hideyasu Sugimoto, Akane Kamiya

**Saitama Cardiovascular and Respiratory Center**

Takashi Ishiguro, Taisuke Isono, Shun Shibata, Yuma Matsui, Chiaki Hosoda, Kenji Takano, Takashi Nishida, Yoichi Kobayashi, Yotaro Takaku, Noboru Takayanagi

**Saitama City Hospital**

Hiroki Tateno, Isano Hase, Shuichi Yoshida, Shoji Suzuki, Miki Kawada, Hirohisa Horinouchi

**Sano Kosei General Hospital**

Takashi Inoue, Takahiro Asami, Toshiyuki Hirano, Keigo Kobayashi, Hatsuyo Takaoka

**Sapporo City General Hospital**

Hisako Sageshima

**Showa University**

Hironori Sagara, Akihiko Tanaka, Shin Ohta, Tomoyuki Kimura

**Showa University Koto Toyosu Hospital**

Naota Kuwahara, Akiko Fujiwara, Tomohiro Matsunaga, Yoko Sato, Takenori Okada

**Tachikawa Hospital**

Hidefumi Koh, Tadashi Manabe, Yohei Funatsu, Fumimaro Ito, Takahiro Fukui, Keisuke Shinozuka, Sumiko Kohashi, Masatoshi Miyazaki

**Toho University Ohashi Medical Center**

Hiroto Matsuse, Norio Kodaka, Chihiro Nakano, Takeshi Oshio, Takatomo Hirouchi

**Tohoku University**

Mitsuhiro Yamada, Koji Murakami, Hisatoshi Sugiura, Hirohito Sano, Shuichiro Matsumoto, Nozomu Kimura, Yoshinao Ono, Hiroaki Baba

**Tokai University**

Koichiro Asano, Tsuyoshi Oguma, Yoko Ito

**Tokyo Institute of Technology**

Takafumi Ueno

**Tokyo Medical and Dental University**

Ryuji Koike, Kunihiko Takahashi, Tatsuhiko Anzai, Satoshi Ito, Akifumi Endo, Yuji Uchimura, Yasunari Miyazaki, Takayuki Honda, Tomoya Tateishi, Shuji Tohda, Naoya Ichimura, Kazunari Sonobe, Chihiro Tani Sassa, Jun Nakajima, Masumi Ai, Akinori Kimura, Takanori Hasegawa, Satoru Miyano

**Tokyo Medical University Hospital**

Shinji Abe, Yuta Kono, Yuki Togashi, Hiroyuki Takoi, Ryota Kikuchi

**Tokyo Medical University Ibaraki Medical Center**

Tomoo Ishii

**Tokyo Metropolitan Police Hospital**

Masayuki Kanai, Tomonori Imamura, Tatsuya Yamashita

**Tokyo Saiseikai Central Hospital**

Ayumi Yoshifuji, Kazuto Ito, Saeko Takahashi, Kota Ishioka, Morio Nakamura

**Tokyo Women’s Medical University**

Etsuko Tagaya, Masatoshi Kawana, Ken Arimura

**Tokyo Women’s Medical University Medical Center East**

Tomohisa Shoko, Mitsuaki Kojima, Tomohiro Adachi, Motonao Ishikawa, Kenichiro Takahashi

**Tosei General Hospital**

Yoshikazu Mutoh, Tomoki Kimura, Tomonori Sato, Reoto Takei, Satoshi Hagimoto, Yoichiro Noguchi, Yasuhiko Yamano, Hajime Sasano, Sho Ota

**Toyohashi Municipal Hospital**

Tomoya Baba, Yasutaka Fukui, Mitsuru Odate, Shuko Mashimo, Yasushi Makino

**Tsukuba Kinen General Hospital**

Hiroko Watanabe

**Uji-Tokushukai Medical Center**

Yusuke Chihara, Mayumi Takeuchi, Keisuke Onoi, Jun Shinozuka, Atsushi Sueyoshi

**University of Tsukuba**

Yoshiaki Inoue, Shigeru Chiba, Kunihiro Yamagata, Yuji Hiramatsu, Hirayasu Kai

**Yamagata University**

Masafumi Watanabe, Sumito Inoue, Akira Igarashi, Masamichi Sato

**Yokohama City University**

Koji Okudela

**Yokohama Municipal Citizen’s Hospital**

Hiroyuki Hayashi, Yukihiro Yoshimura, Natsuo Tachikawa
